# A Preliminary Study of PSMA Fluorescent Probe for Targeted Fluorescence Imaging of Prostate Cancer

**DOI:** 10.3390/molecules27092736

**Published:** 2022-04-24

**Authors:** Haoxi Zhou, Yachao Liu, Xiaojun Zhang, Kuang Chen, Yuan Li, Xiaodan Xu, Baixuan Xu

**Affiliations:** 1 Chinese PLA Medical School, Chinese PLA General Hospital, Beijing 100853, China; drhx_chow@163.com (H.Z.); chenkuang301@163.com (K.C.); 2 Department of Nuclear Medicine, Chinese PLA General Hospital, Beijing 100853, China; yachao301@163.com (Y.L.); plazxj@163.com (X.Z.); xuxiaodan301@163.com (X.X.); 3 Department of Nuclear Medicine, Peking University First Hospital, Beijing 100034, China; yuanli19970115@163.com

**Keywords:** PSMA, radical prostatectomy, fluorescence imaging, positive surgical margin (PSM)

## Abstract

**Purpose:** With the increasing detection rate of early prostate cancer (PCa), the proportion of surgical treatment is increasing. Surgery is the most effective treatment for PCa. Precise targeting of tumors during surgery can reduce the incidence of positive surgical margins (PSMs) and preserve the neurovascular bundles (NVBs) as much as possible. The objective of this study was to synthesize a PSMA fluorescent probe (PSMA-Cy5) and verify the targeting specificity of the probe for prostate cancer, thereby providing a theoretical basis for the development of PSMA fluorescent probes for clinical application in the future. **Methods:** In this study, a novel water-soluble 3H-indocyanine-type bioluminescent dye-Cy5-labeled prostate-specific membrane antigen (PSMA) ligand (PSMA-Cy5) was synthesized by liquid phase synthesis. The PSMA ligand was developed based on the glutamine-urea-lysine (Glu-urea-Lys) structure. The new fluorescent probe was evaluated in vitro and in vivo, and its safety was evaluated. Confocal microscopy was used to observe the binding uptake of PSMA-Cy5 with PSMA (+) LNCaP cells, PSMA (-) PC3 cells and blocked LNCaP cells. In in vivo optical imaging studies, the targeting specificity of PSMA (+) 22Rv1 tumors to probe binding was validated by tail vein injection of PSMA-Cy5. The safety of the PSMA-Cy5 probe was evaluated by histopathological analysis of mouse organs by a single high-dose tail vein injection of PSMA-Cy5. **Results:** In vitro fluorescence cell uptake experiments showed that the binding of PSMA-Cy5 to LNCaP cells has targeting specificity. PC3 cells and blocked LNCaP cells showed almost no uptake. The results of in vivo optical imaging studies showed that the tumor-to-background ratio in the 22Rv1 group was 3.39 ± 0.47; in the 22Rv1 blocking group it was 0.78 ± 0.15, and in the PC3 group it was 0.94 ± 0.09, consistent with the in vitro results. After a high-dose injection of PSMA-Cy5, there were no abnormalities in the tissues or organs of the mice. The probe showed good safety. **Conclusions:** PSMA-Cy5 is a probe with good targeting specificity and low toxicity that can accurately visualize tumors in vivo. This study has an important reference value for the development of PSMA fluorescent probes. In the future, it can be applied to precise tumor imaging during radical prostatectomy to reduce the incidence of postoperative PSM.

## 1. Introduction

PCa is the second most common cancer in men worldwide. In European and American countries, it is the most common malignant tumor in men and seriously threatens men’s health [1,2]. With the continuous development of modern diagnostic techniques, especially the development of specific nuclear medicine molecular imaging, the detection rate of early prostate cancer has been increasing. PSMA is a type II membrane protein that is expressed at low levels on the membranes of normal prostate adenosine cells, while its density and activity are greatly upregulated in prostate cancer cells. As a result, PSMA has rapidly become a specific target for the development of multiple tracers for PET assessment of prostate cancer [3]. Currently, ligands targeting PSMA can be labeled with ^68^Ga, ^18^F, ^99m^Tc, and other isotopes. The most commonly used PSMA tracers in clinical practice are ^18^F-DCFPyL and ^68^Ga-PSMA-617, which can rapidly bind to the PSMA receptor on prostate cancer cells for imaging [4]. Therefore, the application of molecular imaging technology has unique advantages in the early diagnosis of PCa.

Molecular imaging is used for not only diagnosis but also intraoperative navigation. Currently, molecular imaging technologies that can be applied to intraoperative navigation include optics, MRI, ultrasound, and in particular, optical molecular imaging technology, which has been clinically transformative in the operation of breast cancer, glioma, liver cancer and other tumors [5,6]. The most commonly used NIR-I dyes include ICG, IRDye800CW and Cy fluorescent dyes [7,8]. For example, Cy5-labeled matrix metalloproteinases (MMPs) are used for breast cancer surgical navigation, and IRDye800CW-labeled EGFR is used for lymph node tracer surgical navigation. [9,10,11,12]. At present, laparoscopic radical prostatectomy (LRP) is the main method for the treatment of PCa. Compared with open surgery, it has the advantages of less trauma, less bleeding, and clear anatomical structure [13]. Surgical treatment can improve the patient’s condition and cure the disease, but at the same time, it can also cause damage to the body. Urinary incontinence and erectile dysfunction are the most common clinical postoperative complications and seriously affect the quality of life of patients after surgery. At the same time, PSM has always been one of the focuses of attention. It is generally believed that PSM means that there is no guarantee of a sufficient range of resection, which may lead to residual tumor cells, which is a high-risk factor for postoperative biochemical recurrence. Thus, surgery puts forward the concept of “three consecutive victories” in radical prostatectomy, including tumor control, urinary function recovery, and sexual function rehabilitation [14,15]. It is currently believed that the NVB is composed of nerves and blood vessels widely distributed around the prostate and plays key roles in sexual function and urinary control. Therefore, in principle, NVB damage should be avoided as much as possible during surgery to better control the long-term complications of radical prostatectomy [16]. However, too much reserved NVB may cause PSM. Therefore, we hope to use molecular imaging technology to accurately navigate the surgery. By preparing high-sensitivity, high-specificity, and highly safe targeted probes, the tumor can be accurately visualized during the operation, and the tumor boundaries can be defined. Intraoperative planning of surgical methods, timely treatment of tumor residues, reduction of the incidence of PSM, and preservation of NVB as much as possible can reduce the incidence of postoperative urinary incontinence and erectile dysfunction [17]. Cy5 (Cyanine 5) is a far-red fluorescent anthocyanin dye that can be used to label proteins, antibodies, peptides and nanoparticles. We obtained PSMA-Cy5 by labeling the PSMA ligand with Cy5. The goal of this study was to explore the function and use of PSMA-Cy5 as a targeting probe through in vitro and in vivo studies.

## 2. Materials and Methods

### 2.1. General

All chemicals were obtained from commercial suppliers and were used without further purification. Cy5-NHS, OtBu, DCM, DIEA and other probe synthesis reagents were purchased from Chinapeptides Co., Ltd. (Suzhou, Jiangsu, China). RPMI-1640 medium, FBS and P/S were purchased from Gibco Life Technologies (Grand Island, NY, USA). 2-PMPA was purchased from Sigma-Aldrich (Saint Louis, MO, USA). MeOH and DMSO were purchased from Macklin Biochemical Co., Ltd. (Shanghai, China).

### 2.2. Synthesis of PSMA-Cy5

(1) One gram each of H_2_N-Lys(Z)-OtBu and DIEA (2 eq) were dissolved in a 250-mL single-port flask with 50 mL of DCM and stirred at room temperature for 10 min. (2) DIEA (2 eq) was dissolved in a 250-mL single-port flask with 50 mL of DCM and stirred at room temperature for 10 min. (3) Triphosgene (0.33 eq) was added under the protection of N_2_, kept at 0 °C, and stirred for 3 h. (4) H_2_N-Glu(OtBu)-OtBu was added, and after the reaction slowly rose to room temperature, the mixture was stirred overnight until the reaction was complete. (5) The solvent was removed by distillation under reduced pressure and subjected to aqueous extraction with EA and saturated brine 2–3 times, and the organic phase was then distilled under reduced pressure to obtain a crude product. (6) The above crude product was dissolved with EA in a 100-mL single-port flask, and 10% Pd/C, H_2_ was added at room temperature overnight. (7) The reaction was detected by HPLC, Pd/C was removed, and the product was obtained by distillation under reduced pressure. Intermediate A was obtained by preparation. (8) Intermediate A was dissolved in a 250-mL single-port flask with DCM, 1eq Cy5-NHS was added, along with TETN adjusted to pH = 8–9, and the mixture was stirred at room temperature in the dark for 2 h. The solvent was distilled off under reduced pressure. (9) Lysis solution (TFA 95%, EDT 2%, TIS 2%, H_2_O 1%) was added to the flask and shaken on a shaker at room temperature in the dark for 2 h. The lysis solution was dried with nitrogen as much as possible, and a 10-fold volume of ether was added for separation, and the solution was centrifuged. The supernatant was removed, the precipitate was washed with ether six times, and then the product was evaporated to dryness at room temperature to obtain the crude product. (10) The peptide was purified by HPLC and freeze-dried to obtain the finished product. A small amount of finished peptides were subjected to MS and HPLC analysis and identification. The lyophilized peptides were sealed, protected from light and stored at −80 °C.

### 2.3. HPLC Purity Identification and MS Analysis

The purity of the synthesized product PSMA-Cy5 was identified by reverse-phase HPLC (Shimadzu LC-20A, Shimadzu, Japan). The UV absorption spectrum was detected at 220 nm, and the chromatographic column was a C18 reverse chromatographic column (5 μm, 250 × 4.6 mm). The mobile phase ranged from 35% solvent A (0.1% TFA in acetonitrile) and 65% solvent B (0.1% TFA in water) to 65% solvent A and 35% solvent B for a total of 20 min. The product was analyzed by mass spectrometry (MS) (Waters ZQ 2000, Manchester, UK) to determine its molecular weight (Capillary (KV): ±(2500~3000), Desolvation (L/h): 800, Desolvation Temp: 450 °C, Cone (V): 30~50, Run Time: 1 min).

### 2.4. The Binding Affinity of PSMA-Cy5 and PSMA

NAALADase assay [18,19,20,21,22]—The temperature of the incubator was set to 37 °C. The recombinant human PSMA protein was diluted to 0.4 mg/mL with HEPES buffer for use. The NAAG was also diluted with HEPES buffer to 160 µM for use. The probe PSMA-Cy5 was diluted with HEPES buffer to different concentrations: 400 µM, 40 µM, 4 µM, 400 nM, 40 nM, 4 nM, 400 pM, and 4 pM. Then, 25 µL of NAAG, 25 µL of PSMA-Cy5 probe and 50 µL of PSMA recombinant protein were added to the EP tube, which was then centrifuged. After the solution reached the bottom, it was incubated at 37 °C for 1 h. Three parallel reactions were set up for each group. The mixed solution was transferred to a 95 °C metal bath to terminate the denaturation of the protein. Then, 100 µL of OPA detection reagent was added to each tube, and the solution was then mixed well, avoiding light. One hundred microliters of the mixed solution were removed from each tube to a 96-well plate, and the plate was immediately detected on a microplate reader (Ex/Em = 350/450 nm, gain 100).

### 2.5. Excitation and Emission Spectra

The excitation and emission spectra of PSMA-Cy5 were detected and recorded on a Shimadzu RF-6000 spectrofluorometer (Shimadzu, Japan). Four different solvents (DCM, MeOH, DMSO, PBS) were used to prepare a PSMA-Cy5 solution with a concentration of 1 µmol/L. The excitation spectrum was scanned from 500 nm to 700 nm, the data interval was 0.5 nm, and the scanning speed was 600 nm/min. The emission spectrum was scanned from 500 nm to 800 nm, the data interval was 0.5 nm, the scanning speed was 600 nm/min, and the excitation light wavelength was the maximum excitation wavelength measured for each solvent group.

### 2.6. The Absolute Quantum Yield, Photostability and Molar Absorption Coefficient of PSMA-Cy5

The absolute quantum yields (Φ, %) of PSMA-Cy5 in DCM or PBS solutions (1.25 μM) were recorded on a Hamamatsu Quantaurus-QY C11347-11 absolute quantum yield spectrometer (Hamamatsu, Japan). The photostability of PSMA-Cy5 (PBS 5 μM) was performed with constant illumination over 30 min by using a Varian Cary Eclipse fluorescence spectrophotometer (Palo Alto, CA, USA) under the best emission (659.5 nm) and excitation (640.0 nm) wavelength. Absorbance spectra were recorded using a Shimadzu UV-2450 UV-vis spectrophotometer (Shimadzu, Japan). PBS solutions (3 mL) of the probes with different concentrations (1–5 µM) were prepared for measuring the UV-visible data including maximum absorption wavelength (*λ*_abs_, nm) and molar absorption coefficient (ε, L/mol/cm).

### 2.7. Cell Lines and Culture Conditions

The human prostate cancer cell lines LNCaP, 22Rv1 and PC3 were purchased from GuYan Biotech Co., Ltd. (Shanghai, China). LNCaP cells, 22Rv1 cells and PC3 cells were cultured in RPMI-1640 medium in an incubator containing 5% CO_2_ at 37 °C and supplemented with 10% FBS and 1% P/S.

### 2.8. In Vitro Fluorescence Imaging of PSMA-Cy5

Both LNCaP cells and PC3 cells were grown in T25 cell culture flasks (Corning Inc., Corning, NY, USA). The two kinds of cells were seeded in a confocal dish and placed in a 37 °C/5% CO_2_ incubator for 24 h. The medium was changed 2 h before the cell uptake experiment. The medium was aspirated, the cells were rinsed with PBS twice, and 5 μg of PSMA-Cy5 (100 μL) was added to each dish and make up the medium to 2 mL, and the dishes were then incubated in an incubator for 60 min. In the blocking group, LNCaP cells were pretreated with 2-PMPA [23] for 30 min, and then PSMA-Cy5 was added and incubated for 60 min. The procedure was the same as before. The medium with PSMA-Cy5 was aspirated, the cells were rinsed four times with PBS, and then 450 μL of 4% paraformaldehyde was added to fix the cells for 10 min. The fixative was then aspirated, the cells were rinsed with PBS four times, and 450 μL of hoechst33342 stain was added. The stain was aspirated after 10 min, the cells were rinsed with PBS four times, 500 μL of PBS was added, and the cells were observed under a confocal microscope. The cells were washed gently with PBS, avoiding light during the entire procedure.

### 2.9. Animal Model

All animal procedures were conducted in accordance with the protocols approved by the Animal Care and Use Committee of the PLA General Hospital. BALB/c male nude mice (3–4 weeks old, weight 13–15 g) were purchased from Charles River Laboratories (Beijing, China). One hundred microliters of 22Rv1 cell suspension (containing approximately 5 × 10^6^ cells) were subcutaneously injected into the right shoulder of the mouse near the armpit to form a 22Rv1 tumor xenograft. The long and short diameters of the tumor were measured with a Vernier caliper. The tumor volume was estimated according to the formula (V = 1/2 × long diameter×short diameter^2^). When the tumor volume reached 200–300 mm^3^, fluorescence imaging was performed in vivo and in vitro. PC3 cells were cultured in the same way as 22Rv1 cells.

### 2.10. In Vivo and Ex Vivo Fluorescence Imaging

In vivo fluorescence imaging was performed using the French Biospace Lab in vivo optical imaging system, and PHOTON IMAGER OPTIMA multidimensional real-time awake animal in vivo optical imaging system software was used for analysis. A Cy5 exclusive filter was used to collect the PSMA-Cy5 fluorescence data. All images were collected using the same parameters (lamp voltage, filter, exposure height, exposure intensity, exposure time, etc.). The collected images were processed in a unified manner, and the number of photons per second per centimeter square per steradian (ph/s/cm^2^/sr) was used for analysis. The 22Rv1 tumor-bearing mice in the nonblocking group (n = 5) and PC3 tumor-bearing mice (n = 5) were injected with PSMA-Cy5 (0.5 mg/kg) through the tail vein, and fluorescence was measured at 30 min, 60 min, and 120 min after the injection. The blocking group of 22Rv1 tumor-bearing mice (n = 5) was injected with a mixture of 2-PMPA (2 mg/kg) and PSMA-Cy5 (0.5 mg/kg), and fluorescence imaging was performed 60 min after injection. Another nine 22Rv1 tumor-bearing mice were divided into three groups (3/group), PSMA-Cy5 (0.5 mg/kg) was injected into the tail vein, and the mice were euthanized at 30 min, 60 min, and 120 min after injection. The tumors, tissues, and organs were dissected, and a fluorescence imaging system was used for in vitro imaging. The ROI was delineated, and the average fluorescence intensity value was measured.

### 2.11. Acute Toxicity Test

Male ICR mice (3–4 weeks old, 12–16 g) were purchased from Charles River Laboratories (Beijing, China). A large dose of PSMA-Cy5 (100 mg/kg) was injected into the tail vein. After 7 days, the mice were euthanized, and mouse organs (heart, liver, spleen, lung, kidney) were taken, fixed with formalin, dehydrated, paraffin-embedded, sectioned, HE stained and mounted for histopathological analysis. The control group was injected with the same volume of normal saline.

### 2.12. Data Processing and Statistical Analysis

All measured values are expressed as the mean ± standard deviation. The collected measurement values were tested for normality. Two independent sample *t*-tests were used for statistical analysis when they were in line with a normal distribution. *p* < 0.05 was statistically significant. To clarify the tumor contrast, the region of interest was delineated, the average fluorescence intensity of the tumor tissue (T) and the background (B) was collected, the ratio of tumor divided by the background (TBR) was used to express the tumor-background contrast, and the left shoulder near the armpit was selected as the background.

## 3. Results

### 3.1. Synthesis of PSMA-Cy5

**Figure 1:**
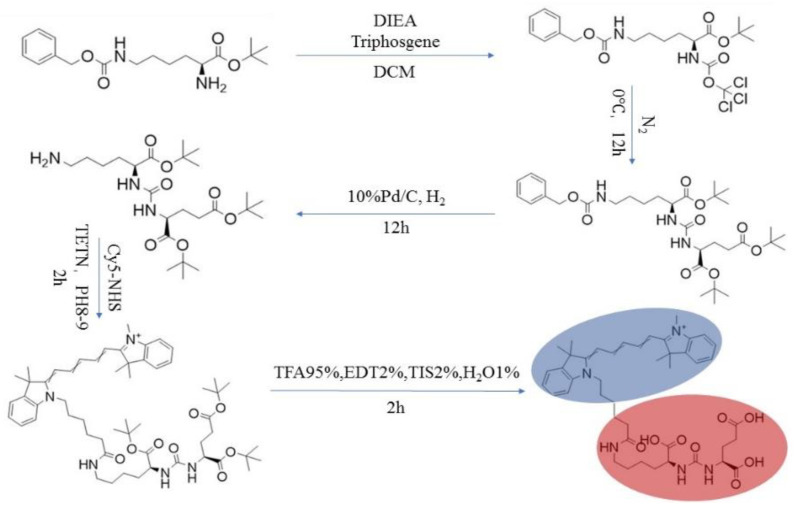
PSMA-Cy5 synthesis route map.

The synthetic steps of PSMA-Cy5 are shown in Figure 1.

### 3.2. HPLC Purity Identification and MS Analysis

**Figure 2:**
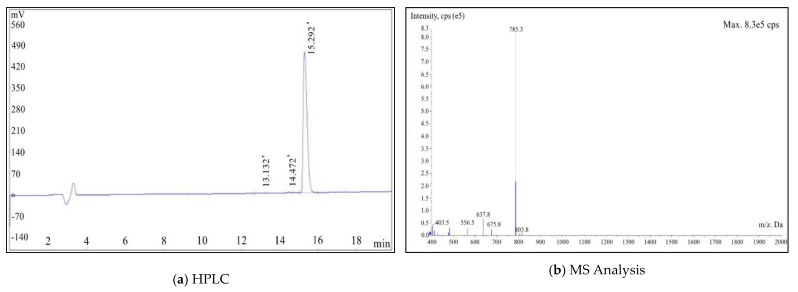
HPLC (**a**) and MS (**b**) analysis results of PSMA-Cy5.

The HPLC purity analysis is shown in Figure 2a, and the purity of the synthesized product was 99.34%. The MS analysis result is shown in Figure 2b, and the molecular weight (M + H^+^) was 785.3.

### 3.3. The Binding Affinity of PSMA-Cy5 and PSMA

The inhibition constant KI value of PSMA-Cy5 was determined by the NAALADase method. The KI value of PSMA-Cy5 is 9.427 × 10^−9^, which is at the nanomolar level. Overall, PSMA-Cy5 showed a high affinity for PSMA.

### 3.4. Excitation and Emission Spectra

**Figure 3:**
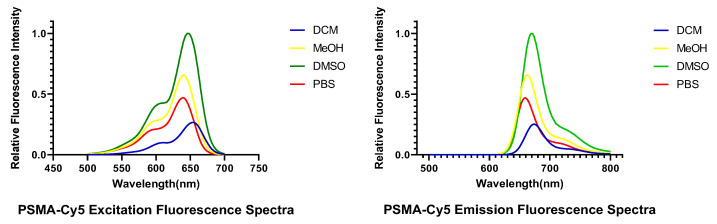
Excitation (**left**) and emission (**right**) spectra of PSMA-Cy5.

The excitation and emission spectra of PSMA-Cy5 are shown in Figure 3. The determination results of the maximum excitation and emission wavelengths of PSMA-Cy5 are shown in Table 1.

### 3.5. The Absolute Quantum Yield, Photostability and Molar Absorption Coefficient of PSMA-Cy5

**Figure 4:**
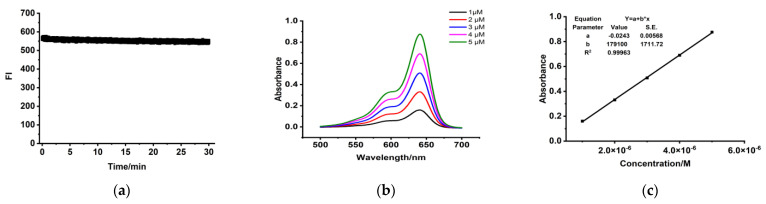
Optical properties determination. (**a**) The photostability of PSMA-Cy5. (**b**) Absorption spectra of PSMA-Cy5 in PBS. (**c**) Linear fitting curves between absorbance and concentration. Molar absorption coefficients (**c**) equal to parameter b.

The absolute quantum yields (Φ, %) of PSMA-Cy5 in DCM and PBS solutions are 31.4% and 12.4%. The photostability of PSMA-Cy5 is shown in Figure 4a. Absorbance spectra and molar absorption coefficient of PSMA-Cy5 are shown in Figure 4b,c.

### 3.6. Binding Specificity of PSMA-Cy5

**Figure 5:**
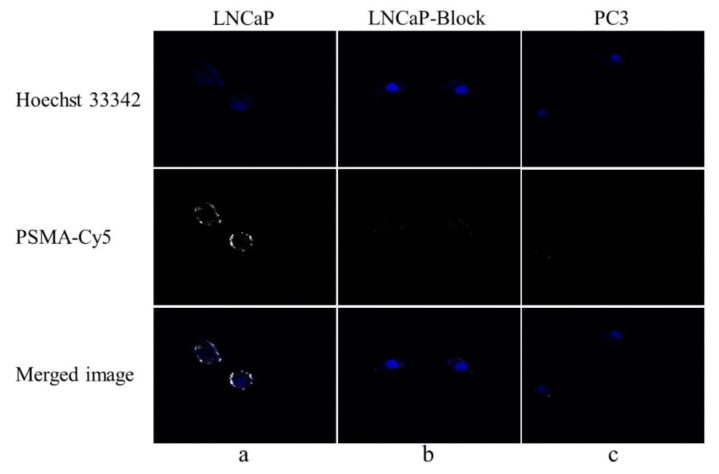
Fluorescence cell uptake experiment. (**a**) PSMA-Cy5 and LNCaP cells were incubated at 37 °C for 60 min. (**b**) After blocking with 2-PMPA for 30 min, PSMA-Cy5 and LNCaP cells were incubated at 37 °C for 60 min. (**c**) PSMA-Cy5 and PC3 cells were incubated at 37 °C for 60 min.

After PSMA-Cy5 and LNCaP cells were incubated for 1 h at 37 °C, fluorescence imaging was performed under a confocal microscope. The LNCaP cell membrane showed a strong fluorescent signal, and the fluorescent signal could also be seen in the cytoplasm, as shown in Figure 5a. There was almost no fluorescence signal in LNCaP cells (Figure 5b) blocked by 2-PMPA and PSMA (-) PC3 cells (Figure 5c). The above results demonstrate that PSMA-Cy5 can specifically bind to the PSMA target on the cell membrane.

### 3.7. In Vivo and Ex Vivo Fluorescence Imaging

**Figure 6:**
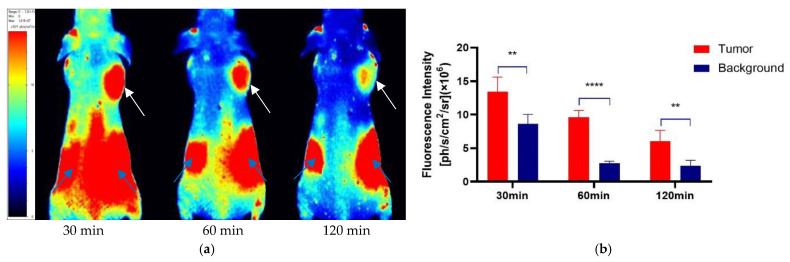
Quantitative analysis of the ROI of in vivo fluorescence imaging. (**a**) Fluorescence imaging of 22Rv1 tumor-bearing mice after tail vein injection of PSMA-Cy5 (0.5 mg/kg). The tumor can be clearly seen at 30 min, 60 min, and 120 min (indicated by white arrows). The fluorescence intensity is recorded as the number of photons per second per centimeter square per steradian (ph/s/cm^2^/sr). (**b**) Comparison and quantitative analysis of PSMA-Cy5 uptake in tumor and background tissues. At each time point, the tumor uptake was significantly higher than the background uptake. Error bars are calculated with standard deviation (n = 5). (**: *p* < 0.01; ****: *p* < 0.0001).

**Figure 7:**
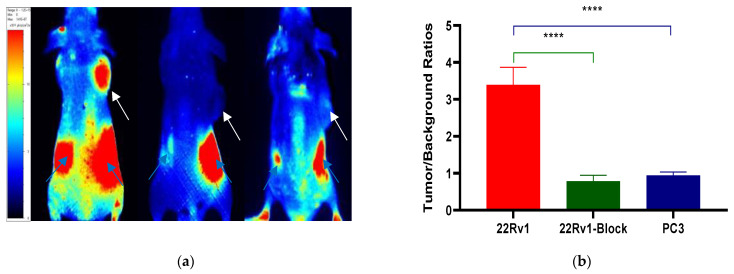
TBR analysis of in vivo fluorescence imaging. (**a**) Fluorescence imaging 60 min after injection of PSMA-Cy5 into the right shoulder of a tumor-bearing mouse (left). After 2-PMPA blockade, tumor uptake of PSMA-Cy5 was measured (middle). Fluorescence imaging of a PC3 tumor-bearing mouse on the right shoulder 60 min after injection of PSMA-Cy5 via the tail vein (right). (**b**) 22Rv1 nonblocking group or blocking group and PSMA (-) PC3 tumors, based on the TBR of the ROI analysis of PSMA-Cy5 uptake at 60 min (****: *p* < 0.0001).

**Figure 8:**
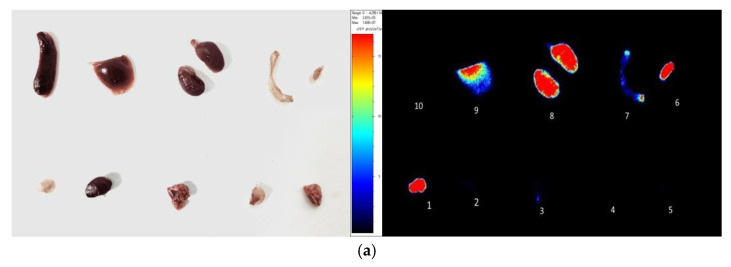

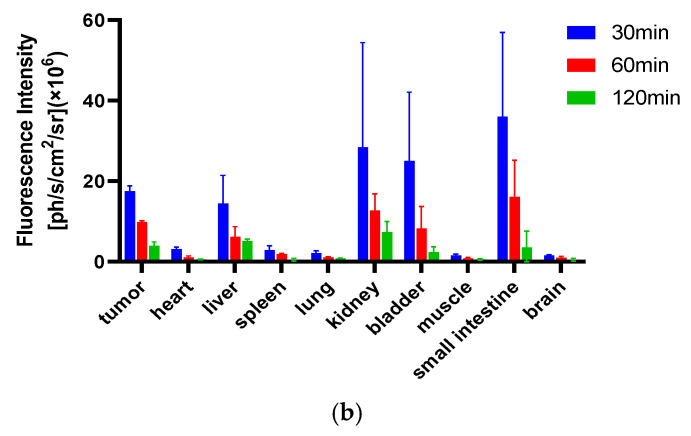
In vitro fluorescence imaging (**a**). Sixty minutes after the injection of PSMA-Cy5, the mice were euthanized and dissected for the in vitro imaging of tumors, tissues and organs; 1, tumor, 2, heart, 3, lung, 4, muscle, 5, brain, 6, bladder, 7, small intestine, 8, kidney, 9, liver, 10, spleen. (**b**) After injection of PSMA-Cy5, ROI analysis of the fluorescence intensity of the main tissues at 30 min, 60 min, and 120 min after injection was performed. Error bars are calculated as standard deviation (n = 3).

After the tail vein injection of PSMA-Cy5 (0.5 mg/kg), fluorescence images of 22Rv1 tumor-bearing mice were collected at different time points (Figure 6a). Thirty minutes after the injection of PSMA-Cy5, the probe showed rapid 22Rv1 tumor targeting in mice. At 60 min after injection, the contrast between the tumor and the background was the highest. A histogram of the average fluorescence intensity of the tumor and background over time was drawn (Figure 6b). As time passed, the fluorescence intensity of the tumor and the background continued to decrease. Compared with the fluorescence intensity of the tumor at 60 min, the fluorescence intensity of the tumor at 120 min decreased. In contrast, the elution of the probe was faster in normal tissues. PSMA (-) PC3 tumor-bearing mice had very low probe uptake by the tumor, which was significantly lower than that in the 22Rv1 group. A blocking experiment was used to verify the specificity of PSMA-Cy5 to PSMA, and the tumor-bearing mice in the blocking group were injected with a mixture of 2-PMPA and PSMA-Cy5. The results show that PSMA blockers can significantly reduce the tumor uptake of probes. The tumor fluorescence intensity of the 22Rv1 tumor-bearing mice after blocking was even lower than that of the PC3 tumor-bearing mice. Figure 6a shows the fluorescence images of the 22Rv1 tumor-bearing mice in the nonblocking and blocking groups and the PC3 tumor-bearing mice 60 min after the injection of PSMA-Cy5. By delineating the ROI, the average fluorescence intensity of the tumor was collected, and the TBR was calculated 60 min after injection. After 2-PMPA blocking, the TBR decreased from 3.39 ± 0.47 to 0.78 ± 0.15 (*p* < 0.05). The TBR of the PC3 tumor-bearing mouse group was 0.94 ± 0.09, which was also significantly lower than that of the 22Rv1 tumor-bearing mouse nonblocking group (*p* < 0.05) (Figure 7b). In summary, PSMA-Cy5 can specifically bind to PSMA, which is consistent with the results of in vitro cell experiments. In addition, the probes were imaged in vitro to analyze the distribution and metabolism of the probes in the body. Nine 22Rv1 tumor-bearing mice were divided into three groups (3/group). After the tail vein injection of PSMA-Cy5 (0.5 mg/kg), the mice were euthanized at 30 min, 60 min, and 120 min after the injection. Tumors, tissues and organs were collected for in vitro imaging (Figure 8a,b). The results show that the probe is mainly metabolized through the liver-intestine and kidney-bladder pathways. In vivo fluorescence imaging also clearly showed that the intestinal tract had extremely high fluorescence (indicated by blue arrows in Figure 6a and Figure 7a), which was consistent with the results of in vitro distribution imaging.

### 3.8. Acute Toxicity Test

**Figure 9:**
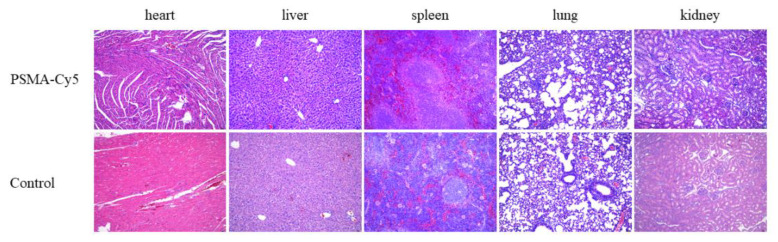
Histopathological analysis of the acute toxicity test. Organs include heart, liver, spleen, lungs, and kidneys. HE staining of mouse organ sections after high-dose injection of PSMA-Cy5 (**top**) and HE staining of mouse organ sections in the control group (**bottom**).

Seven days after the high-dose injection of PSMA-Cy5, mouse organs were taken for histopathological analysis. The results are shown in Figure 9. The organs of the high-dose injection and control groups were normal. No organ damage was seen. Thus, PSMA-Cy5 was demonstrated to have better safety.

## 4. Discussion

In recent decades, optical molecular imaging technology has developed rapidly. It can visualize tumors in vivo with high spatial and temporal resolution and can also be imaged in real time. The use of tumor-specific molecules to modify fluorescent materials has a strong targeting ability, which can significantly increase the TBR between the tumor site and the normal tissue around the tumor and achieve highly specific tumor imaging. Antibodies, peptides and nucleic acid aptamers that target tumors have received extensive attention. Compared with other conjugates, peptides have a high affinity for targets, strong specificity and low toxicity and have good clinical application prospects [24]. ^18^F-DCFPyL is a PSMA-specific small molecule imaging agent developed based on the Glu-urea-Lys short peptide structure. It has a high affinity for prostate cancer, is stable in vivo, has a fast metabolism, and has good biological distribution characteristics [25,26]. Therefore, based on the structure of ^18^F-DCFPyL, we used Cy5 fluorescent dye instead of the nuclide coupling group to label the Glu-urea-Lys active peptide in ^18^F-DCFPyL and obtained the targeted fluorescent imaging agent PSMA-Cy5 for PCa.

We used a liquid phase synthesis method and purified the product to obtain high-purity PSMA-Cy5. Four different solvents were used to configure the PSMA-Cy5 solution, and the excitation and emission spectra of the probe were measured. The results showed that the excitation wavelength peak of PSMA-Cy5 was 640.0–653.5 nm, and the emission wavelength peak was 659.5–674 nm. After Cy5 fluorescent dye is connected to the PSMA ligand, the fluorescence characteristics are essentially unchanged, yielding a relatively stable fluorescent probe. By adopting the NAALADase method, the in vitro affinity determination of PSMA-Cy5 showed that the affinity was on the nanomolar scale, and PSMA-Cy5 had a high affinity for the PSMA recombinant protein. The results of in vitro cell uptake experiments showed that LNCaP cells had high uptake of PSMA-Cy5 and blocked LNCaP cells and PC3 cells had almost no uptake of PSMA-Cy5, which further proved the targeting specificity of PSMA-Cy5. At the same time, PSMA-Cy5 was observed to bind to cell membrane sites under confocal microscopy. The results of in vivo animal imaging are also very impressive. The 22Rv1 tumor-bearing mice in the nonblocking group showed rapid tumor targeting 30 min after injection of the PSMA-Cy5 probe through the tail vein, and the TBR reached its highest value at 60 min. After blocking with a blocker, the tumor uptake of PSMA-Cy5 decreased significantly. After injection of the control group of PC3 tumor-bearing mice, the tumor uptake of PSMA-Cy5 was very low. Based on the above results, in animals, PSMA-Cy5 shows high targeting specificity for prostate cancer, which is consistent with the results of in vitro experiments. The metabolism and distribution of the probe were then analyzed in vivo by in vitro optical imaging. In addition to the high uptake of PSMA-Cy5 by tumors, both the liver and kidney exhibited fluorescence, which was different from the metabolism of ^18^F-DCFPyL. PSMA-Cy5 was metabolized not only by the kidney but also through the liver-intestine metabolic pathway. The uptake of PSMA-Cy5 by the heart, lung, muscle, brain, spleen and other organs was very low, and the tumor-muscle ratio could be as high as 12.19 ± 3.15 at 60 min. In terms of the safety of the PSMA-Cy5 probe, we performed an acute toxicity test, and following a single high-dose injection, it did not cause tissue or organ damage to mice. The results proved the low toxicity and safety of PSMA-Cy5. In summary, fluorescent dye-labeled PSMA ligands are very promising and are fluorescent probes with high specificity.

However, PSMA-Cy5 eluted faster in tumors. Although the elution rate of normal tissue is faster than that of tumors, the time for tumor imaging is enough to complete the operation. However, the location of the prostate is close to the bladder, and the high fluorescence of the bladder may interfere with the imaging. Therefore, we still hope to slow down the elution of the probe from the tumor to minimize the interference of the bladder. We can achieve this by modifying the probe structure, such as by adding a linker or hydrophobic structure or replacing the fluorescent dyes. Based on the results of this research, we will continue to develop ICG, IRDye800CW and other fluorescent dye-labeled PSMA ligands and plan to develop nuclide and fluorescent dual-modal probes with rapid targeting of prostate cancer. In this study, a multifunctional probe that can be used not only for diagnosis but also for navigating surgery was prepared.

## 5. Conclusions

Cy5 was used to label the PSMA ligand (PSMA-Cy5). PSMA-Cy5 had high specificity for PSMA (+) prostate cancer and had low toxicity and high safety. The rapid targeting and high tumor-background contrast of PSMA-Cy5 demonstrated that PSMA ligand-mediated prostate cancer targeted fluorescence imaging has high potential value, with important significance and value in basic scientific research and intraoperative navigation in clinical surgery. A theoretical basis for the future development of PSMA nuclides and fluorescent dual-modal probes that integrate diagnosis and treatment is proposed.

## Figures and Tables

**Table 1:** Excitation and emission spectra of PSMA-Cy5.

Probe	Fluorescence Spectra		
	Solvent	λex/nm	λem/nm
PSMA-Cy5	DCM	653.5	674.0
	MeOH	640.5	662.5
	DMSO	646.0	669.5
	PBS	640.0	659.5

## Data Availability

All data generated or analyzed during this study are included in this published article.

## Sample Availability

Sample of the compound PSMA-Cy5 are available from the authors.

## Abbreviations

PSMA	Prostate-specific membrane antigen
PSM	Positive surgical margin
NVB	Neurovascular bundle
Glu	Glutamate
Lys	Lysine
OtBu	Oxytert-butyl
DIEA	Diisopropylethylamine
DCM	Dichloromethane
EA	Ethyl acetate
HPLC	High-performance liquid chromatography
NHS	*N*-Hydroxysuccinimide
TETN	Triethylamine
TFA	Trifluoroacetic Acid
TIS	Triisopropylsilane
EDT	Ethanedithiol
PBS	Phosphate-buffered saline
OPA	O-Phthaldialdehyde
HEPES	*N*-2-hydroxyethylpiperazine-*N*-ethane-sulfonic acid
NAAG	*N*-acetyl-aspartyl-glutamate
MeOH	Methanol
DMSO	Dimethyl sulfoxide
FBS	Fetal bovine serum
ROI	Region of interest
PET	Positron emission tomography
CT	Computed tomography
^18^F-FDG	^18^F-Fluorodeoxyglucose
ICG	Indocyanine Green
